# Isobar hybrid dynamic stabilization with posterolateral fusion in mild and moderate lumbar degenerative disease

**DOI:** 10.1186/s12891-023-06329-6

**Published:** 2023-03-23

**Authors:** Jianbin Guan, Tao Liu, Xing Yu, Ningning Feng, Guozheng Jiang, Wenhao Li, He Zhao, Yongdong Yang

**Affiliations:** Dongzhimen Hospital, Beijing University of Chinese Medicine, Haiyuncang No.5, Beijing, 100007 China

**Keywords:** Lumbar degenerative disease, Hybrid dynamic stabilization and fusion, Isobar TTL, Posterolateral fusion

## Abstract

**Objective:**

The aim of this study was to investigate the feasibility of using the Isobar TTL system and posterolateral fusion in a two-segment hybrid fixation approach, combined with spinal decompression, for treating mild and moderate lumbar degenerative disease. Specifically, we sought to evaluate the effectiveness of this approach for managing two-segment mild and moderate lumbar degenerative disease, and to determine whether it could provide a safe and reliable alternative to traditional surgical methods.

**Methods:**

This retrospective study included 45 consecutive patients with two-level lumbar disc herniation or spinal stenosis, 24 of whom underwent the TTL system and posterolateral fusion combined (TTL group), and 21 of whom underwent posterolateral fusion alone (Rigid group). The surgical segment, admission diagnosis, operation time, and intraoperative bleeding were recorded separately for the two groups of patients. Imaging studies included pre- and postoperative radiography, magnetic resonance imaging, and computed tomography. The clinical outcomes were measured by Oswestry Disability Index (ODI) scores, and a visual analogue scale (VAS) for back and leg pain.

**Results:**

All patients completed the surgery successfully with a mean follow-up of 56.09 months. The operative time and intraoperative bleeding were lower in the TTL group than in the Rigid group (*p* < 0.05). All patients showed significant improvements in clinical outcomes, including VAS for back and leg pain, and ODI scores (*p* < 0.05). ODI scores, the TTL group was better than the Rigid group at 1 year after surgery and at the final follow-up (*p* < 0.05). Postoperative surgical segment range of motion (ROM) decreased in both groups (*p* < 0.05). The postoperative ROM of the upper adjacent segment increased in both groups and was significantly higher in both groups at the last follow-up compared with the preoperative period (*p* < 0.05), and the superior adjacent segment rom of the TTL group was lower than the Rigid group (*p* < 0.05). The modified Pfrrmann classification of the superior adjacent segment was significantly increased in both groups at the last follow-up (*p* < 0.05). And in the TTL group, ROM, DH, and modified Pfrrmann grading of dynamic segment outperformed fusion segments. According to the UCLA classification, the incidence of adjacent segment degeneration (ASD) was 4.2% in the TTL group and 23.8% in the Rigid group, and the incidence of ASD was lower in the TTL group than in the Rigid group (*P* < 0.05).

**Conclusion:**

The Isobar TTL System was utilized in two-level lumbar hybrid surgery, resulting in no evident indications of lumbar instability being detected on X-rays captured at a minimum of 4 years after the operation, while retaining partial range of motion of the surgical segment. The general clinical efficacy is equivalent to titanium rod fusion surgery, presenting an alternative treatment for individuals with mild and moderate lumbar degenerative disease.

## Introduction

Spinal fusion surgery, which relieves symptoms by decompressing the nerve root compression of the operated segment and reconstructs the stability and sequence of the operated segment, has been thought to be the standard treatment for patients with lumbar degenerative disease. However, fusion changes the normal biomechanical environment of the functional units of the lumbar spine, resulting in loss of motion of the surgical segment and accelerated degeneration of the adjacent segment by stress concentration [1, 2]. Additionally, the fusion of multiple spinal segments may result in additional issues, such as stiffness in the low back and persistent pain at the site of the cancellous bone graft donor [3]. Due to the numerous issues with fusion, the idea of non-fusion and dynamic fixation emerged in the 1980s. This resulted in the development of several new devices, including the posterior dynamic stabilization system based on the pedicle screw, the posterior interspinous bracing device, and the interlaminar bracing device [2, 4]. In order to avoid the drawbacks associated with fusion and rigid fixation, the Isobar TTL semi-rigid dynamic stabilization system is one of them. The Isobar TTL system was originally designed for fusion surgery to facilitate intervertebral fusion based on Wolff's law and provides spinal stabilization while preserving relatively constant mobility (± 2.25°) of the surgical segment, and the system is currently being used for single-segment non-fusion procedures with good clinical results [5–7]. However, there have been no reports of the Isobar TTL system being used in combine with posterolateral fusion for two-segment hybrid surgery. Posterolateral fusion is used by our team for both Isobar TTL spinal fusion and titanium rod spinal fusion because it is less disruptive to the facet joint and the physiological environment of the fixed segment than interbody fusion, minimizing the mechanical distribution of the fixed segment.

This study used a retrospective analysis of indicators related to patients who underwent surgical treatment with the Isobar TTL hybrid fixation and titanium rod internal fixation system at our institution from March 2014 to July 2018 to investigate the feasibility of the Isobar TTL system combined with posterolateral fusion for two-segment hybrid fixation combined with spinal decompression for two-segment mild and moderate lumbar degenerative disease.

## Materials and methods

### Patient

Consecutive patients with symptomatic two-segment mildly lumbar degenerative disease who underwent posterior decompression and Isobar TTL hybrid dynamic stabilization (Scient'x-Alphatec, France) and posterolateral fusion, or posterior spinal rigid fixation (Wegortho company, Shandong, China) and posterolateral fusion in Dongzhimen Hospital Beijing University of Chinese Medicine, between 2014 and 2018 were reviewed retrospectively.

#### TTL group

**Fig. 1:**
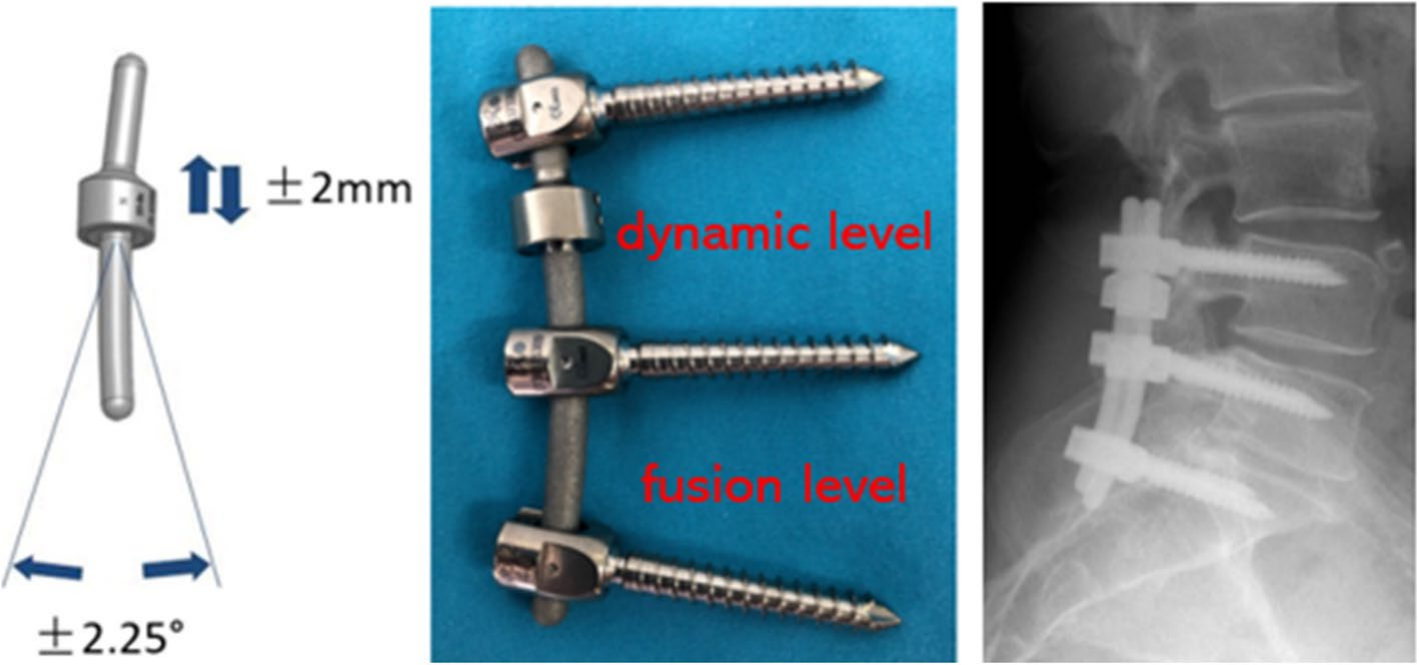
Hybrid Isobar TTL dynamic stabilization and posterolateral fusion system; the rod has the fixed mobility ± 2.25°, and the titanium ring with ± 2 mm longitudinal movement distance

Posterolateral fusion is chosen for segments with severe degeneration, and non-fusion dynamic fixation is chosen for segments with relatively mild degeneration. The severity of segmental degeneration was determined by an experienced radiologist (Zhao He). The severity of segmental degeneration can cause corresponding clinical symptoms in patients, and the classification of severity is relative. For example, if a patient with L4/S1 has degenerative changes in both segments of the lumbar spine, the L5/S1 segment has narrowing of the intervertebral space, calcification of the ligamentum flavum, severe stenosis of the spinal canal, lumbar disc herniation and lumbar instability, while the L4/5 segment has only spinal canal stenosis and disc degeneration, then the L4/5 segment is a less degenerative segment compared to L5/S1 (Figs. 1 and 5).

#### Rigid group

Posterolateral fusion was used for both segments in patients with fusion of titanium rods, regardless of the degree of degeneration.

The clinical presentations of patients in the series included mechanical low back pain, focal radiculopathy, or neurogenic claudication. MRI showing nerve root compression or spinal stenosis in two segments (L3/L5 or L4/S1) with herniated or significantly prolapsed discs (more than half of the spinal canal) or lumbar spondylolisthesis (Meyerding I and II).

Patients were excluded if they had significant anxiety, depression, or other psychological disorders, severe coronary artery disease, cerebral vascular accident, or malignancy, severe lumbar instability (lumbar spondylolisthesis Meyerding III and IV or presence of isthmic fracture), or severe medical conditions. Additionally, patients who were unreachable for follow-up or who failed to complete assessments at each time point were also excluded from data analysis. This study was approved by the institutional ethics committee in Dongzhimen Hospital Beijing University of Chinese Medicine (2022DZMEC-085–04). Every patient freely signed an informed consent form before the operation.

### Surgical technique

All procedures are performed by the same surgical team. Under general anesthesia, the patient was positioned in the prone position with sufficient or acquired lumbar lordosis (Place two pads each on the patient's chest and hips). The paravertebral muscles are exposed layer by layer using the posterior median approach to the lumbar spine, and the paravertebral muscles on both sides are stripped down to the lateral aspect of the bilateral synovial joints while taking care to preserve the joint capsule. Intraoperative fluoroscopy was then applied for surgical segment confirmation. A universal pedicle screw of appropriate length is placed (with the tip of the nail pointing to the upper endplate, as much as possible in one pass to avoid adjusting the nail path). Bone biting forceps are used to remove part of the spinous process and the vertebral plate of the responsible segment, to remove the hyperplastic bone and the hypertrophic ligamentum flavum, and to protect the facet joint. The facet joints were preserved without violation at dynamic stabilization levels, except that the medial one-third of the facets were resected in selected cases to achieve adequate decompression. Decompression of the lateral saphenous fossa is performed by less invasive decompressive (undermining decompressive) until the nerve root canal and central vertebral canal compression is completely relieved, the free prolapsed nucleus pulposus tissue is explored and removed (inclusive protruding discs are not treated if they do not compress the nerve root), and the vertebral space of the operated segment is not harassed as much as possible. After adequate decompression, in the Rigid group, the clipped spinous process and the vertebral plate were trimmed into cancellous bone particles, mixed with allograft bone for posterolateral bone grafting, and the titanium rods were properly bent, with the upper rods attached to the transverse joints and the locking nail tails. In contrast, in the TTL group, dynamic fixation without fusion was carried out in less degenerated segments with locking bolts and posterolateral implantation fusion in severely degenerated segments (Fig. 1). Finally, the wound was flushed, the bleeding was completely stopped, a drainage tube was placed, and the wound was closed layer by layer.

Postoperative antibiotics are given for 24 h to prevent infection, drains are left in place for 24–48 h, and stitches are removed 12–14 days after surgery. And on the first postoperative day, the patient was instructed to perform straight leg elevation to reduce nerve root adhesions. The patient wore a brace to walk on the ground 1 week after surgery. The patient was advised to exercise the lumbar back muscles while on bed rest and to wear the brace for 2 months following surgery.

### Clinical evaluation

Our database was built prospectively with scheduled clinical and radiological examinations for selected patients at each clinical visit. Standard pre- and post-operative questionnaires and clinical evaluations were aimed to be completed at 3 and 12 months after surgery and with a 12-month interval thereafter. The pain scores included visual analogue score (VAS) for back and leg pain, respectively. The functional evaluation included Oswestry Disability Index (ODI) score. All the subjective questions were answered by patients themselves with assistance from our attending physician. The objective assessment was performed by the 2-attending physician under the supervision of the third physicians.

### Radiological evaluation

All patients underwent preoperative standard anteroposterior and dynamic lateral radiographs, magnetic resonance imaging (MRI), and CT scans for evaluation. Postoperative follow-up included both plain and dynamic radiographs at 3 and 12 months after surgery and every 12 months thereafter. Follow-up CT and MRI were undertaken at final follow-up.

**Fig. 2:**
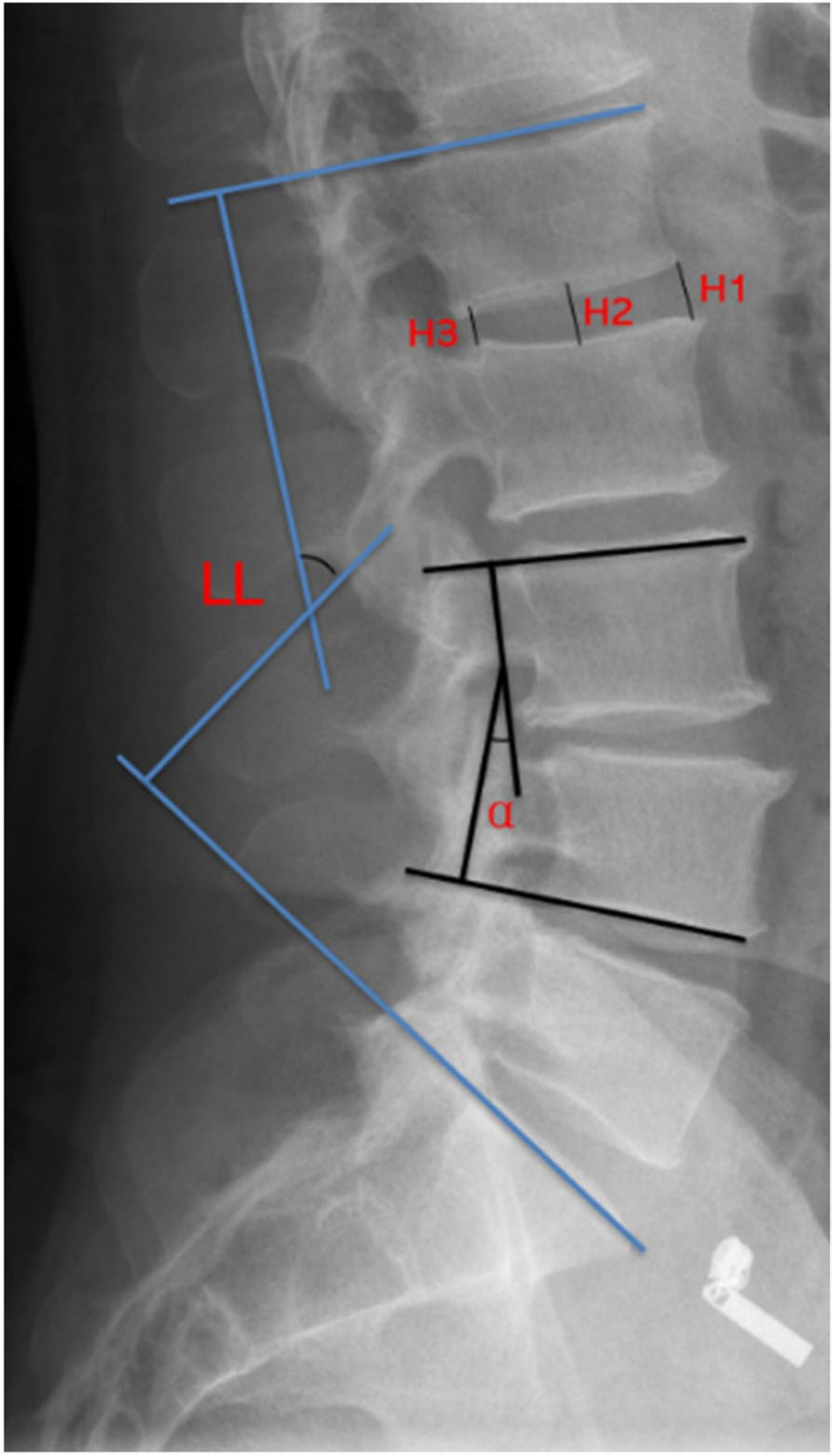
Imaging measurement methods (The range of motion (ROM) of the surgical segment and the adjacent segment is assessed by measured Cobb angle (α) of the surgical segment and adjacent segments on dynamic lateral radiographs. The lumbar lordosis angle (LL) was measured by a standing lateral radiograph. The Cobb angle between upper endplate of L1 and S1 was defined LL

The range of motion (ROM) of the surgical segment and the adjacent segment is assessed by measured Cobb angle (α) of the surgical segment and adjacent segments on dynamic lateral radiographs. The lumbar lordosis angle (LL) was measured by a standing lateral radiograph. The Cobb angle between upper endplate of L1 and S1 was defined LL (Fig. 2).

Mid-sagittal radiographs of the lumbar spine were used to determine the disc height (DH) of the surgical segment and the adjacent segment. At each follow-up visit, the DH was calculated as the average of the anterior, middle, and posterior margins of the intervertebral space on preoperative and postoperative mid-sagitta radiographs *DH* = *H1* + *H2* + *H3/3* (Fig. 2).

Degeneration of the intervertebral disc was assessed using modified Pfrrmann grading on median lateral and transverse MRI T2W1-weighted images [8]. The University of California at Los Angeles (UCLA) grading was also used to evaluate the degeneration of adjacent segments, and the preoperative and final follow-up median sagittal radiographs and CT images were used to evaluate the intervertebral space height, bone formation and the presence of endplate sclerosis as the key evaluation indexes of this system. The combination of the two can provide a comprehensive assessment of disc degeneration. Because adjacent segment degeneration (ASD) is frequently found in the upper adjacent segment of the operated segment, only the upper adjacent segment imaging indices were evaluated in this study [9]. Screw loosening is diagnosed by the "double halo sign" on neutral lateral and oblique radiographs [10].

Two radiologists and two neurosurgeons reviewed the images independently and the coauthors made the final decision if there was any ambiguity among interpretations.

### Statistical analysis

All statistical analyses were performed using IBM SPSS ver. 19.0 (IBM Co., Armonk, NY, USA). Independent t-tests and paired t-tests were used for continuous variables, and the chi-square test was applied for categorical data. A *p*-value of 0.05 was considered to be statistically significant.

## Results

**Fig. 3:**
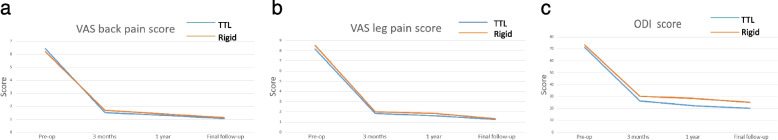
VAS back pain score (**a**), VAS leg pain score (**b**), ODI score (**c**). All scores decreased significantly during the 3 months after surgery and subsequently plateaued. Note: pre-op, 3 months after surgery, 1 year after surgery, final follow-up

**Fig. 4:**
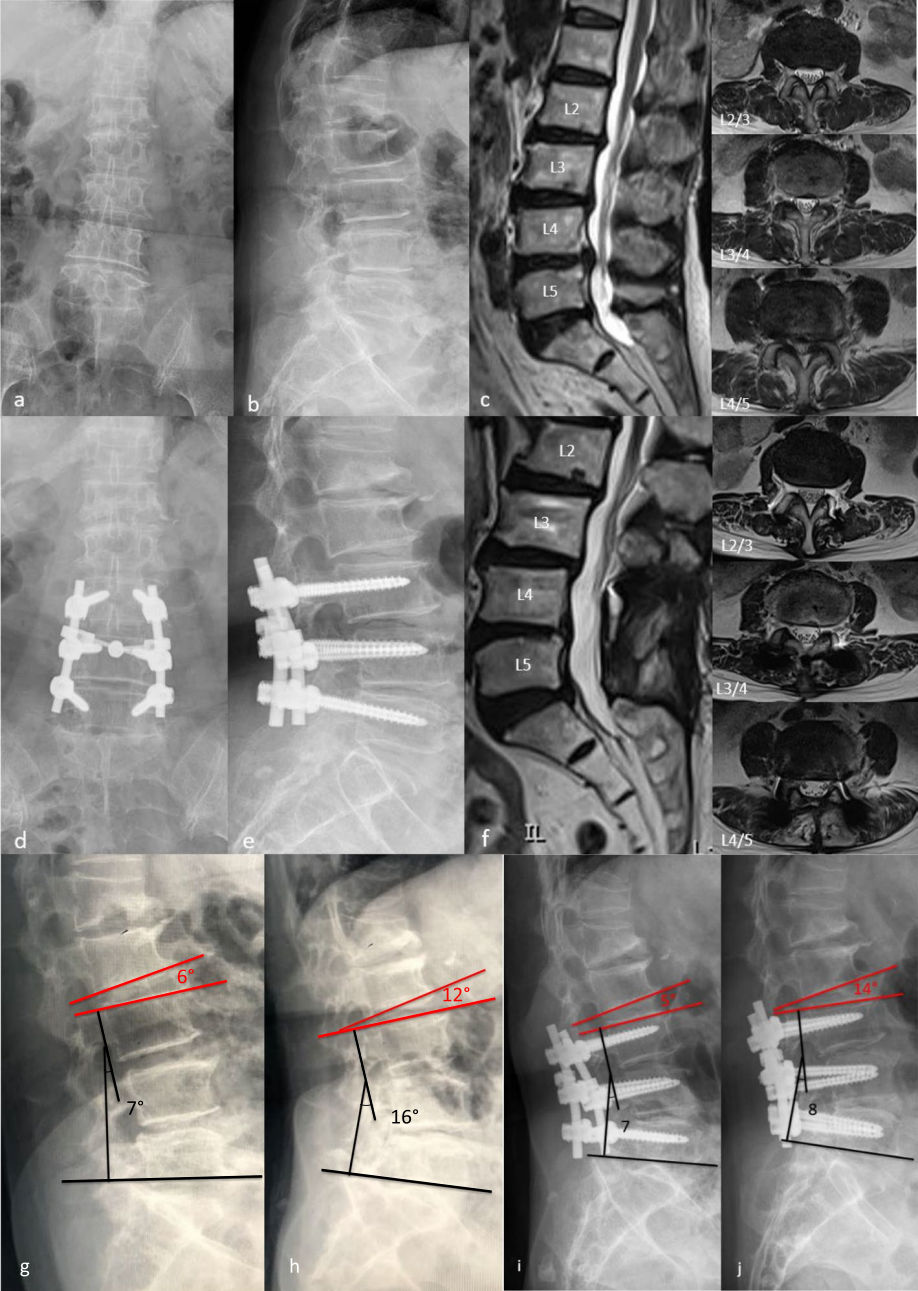
A 63-year-old male patient underwent two-segment rigid fixation with posterolateral fusion (L3/5). **a–c** Preoperative radiological imaging confirmed L4/5 spinal stenosis. **d–f** Radiological imaging 25 months after surgery indicated that satisfactory decompression was achieved. **g-h** Preoperative radiological imaging showed that ROM of upper adjacent segment and operative segment is 6° and 9° respectively. **i-j** Final follow-up radiological imaging showed that ROM of upper adjacent segment and operative segment is 9°and 1° respectively

Forty-five consecutive patients with two-level lumbar disc herniation or spinal stenosis, 24 of whom underwent the TTL system and posterolateral fusion combined (TTL group, Fig. 4), and 21 of whom underwent posterolateral alone (Rigid group, Fig. 3). All patients completed the clinical and radiological evaluations for more than 48-month post-operation. Their mean age was 66.54 ± 5.64 years at the time of surgery; 24 were male and 21 were female patients (Table 1). The mean follow-up duration was 56.09 ± 5.47 months.

**Table 1:** General data of the two group

	TTL (*n* = 24)	Rigid (*n* = 21)	*P* value
Age	65.5 ± 5.79	66.71 ± 5.55	0.478
Gender (male/female)	14/10	10/11	0.472
Follow-up time (months)	54.63 ± 4.32	57.76 ± 6.23	0.054
Operative segment			0.926
L3/5	6	5	
L4/S1	18	16	
Primary diagnosis			0.967
Spinal stenosis	15	13	
Lumbar disc herniation	9	8	
lumbar spondylolisthesis (Grade I and Grade II)	18	20	
Operation time (min)	145.83 ± 20.62	165.24 ± 19.90	0.003*
Intraoperative blood loss (mL)	168.79 ± 59.54	235.23 ± 74.41	0.002*

Values are presented as mean ± standard deviation

*P* values are based on the Independent two‑sample t‑test * or chi-square test†

Of the 45 patients analyzed, a total of 270 screws, 48 Iosbar TTL titanium rods and 42 common titanium rods were inserted in this series. 28 patients had lumbar stenosis and 17 had herniated discs. The distribution of levels varied from L3 through to S1. The operation time of TTL group was shorter than Rigid group (145.83 ± 20.62 min vs. 165.24 ± 19.90 min, *P* = 0.003). The intraoperative blood loss of TTL group was shorter than Rigid group (168.79 ± 59.54 mL vs. 235.23 ± 74.41 mL, *P* = 0.002) (Table 1). No serious intraoperative or postoperative complications such as nerve root injury, dural tear, cauda equina injury, vertebral fracture, etc. were observed in either group, and no broken nails and rods or screws were loosened in either the TTL or Rigid groups after surgery.

### Clinical outcomes

In general, the patients of each group had a significant decrease in pain scores (VAS of back and leg pain) after surgery at each time of post-operation when compared to pre-operation (Fig. 3, Table 2). The VAS scores for back pain of TTL group were 6.42 ± 0.78 before operation and 1.04 ± 0.62 at final follow-up (*p* < 0.05). The VAS scores for leg pain of TTL group were 7.04 ± 1.12 before operation and 1.08 ± 0.58 at final follow-up (*p* < 0.05). The VAS scores for back pain of Rigid group were 6.19 ± 1.08 before operation and 1.14 ± 0.73 at final follow-up (*p* < 0.05). The VAS scores for leg pain of Rigid group were 7.24 ± 1.30 before operation and 1.14 ± 0.57 at final follow-up (*p* < 0.05). Furthermore, there were no significant differences in pain scores (VAS of back and leg pain) between the TTL group and the Rigid group pre-operation and at each post-operative time.

**Table 2:** ODI and VAS values

	Pre-operation	3 months after operation	1 year after operation	Final follow-up	*P* value
VAS (Back)					
TTL	6.42 ± 0.78	2.08 ± 0.53	1.50 ± 0.59	1.04 ± 0.62	0.000
Rigid	6.19 ± 1.08	2.01 ± 0.49	1.52 ± 0.51	1.14 ± 0.73	0.000
*P* value	0.419	0.813	0.886	0.618	
VAS (Leg)					
TTL	7.04 ± 1.12	2.29 ± 0.46	1.63 ± 0.49	1.08 ± 0.58	0.000
Rigid	7.24 ± 1.30	2.24 ± 0.44	1.80 ± 0.51	1.14 ± 0.57	0.000
*P* value	0.589	0.693	0.226	0.732	
ODI%					
TTL	71.17 ± 9.41	27.67 ± 4.56	24.67 ± 2.68*	23.08 ± 3.12*	0.000
Rigid	73.16 ± 9.03	30.19 ± 4.64	28.19 ± 3.84	28.57 ± 3.36	0.000
*P* value	0.474	0.073	0.001	0.000	

Data are presented as mean ± standard deviation

^*^Significant difference at the same time point between the TTL and Rigid groups using the Independent two‑sample t‑test, *P* < 0.05

The patients of each group in the present series had satisfactory improvement after surgery. The ODI scores, improved significantly after surgery at 48-month post-operation when compared to pre-operation. Furthermore, 1 year after surgery and at the final follow-up, the ODI scores of the TTL group were lower than those of the Rigid group. The ODI scores between TTL group and Rigid group at 1 year after surgery were 24.67 ± 2.68 vs. 28.19 ± 3.84 (*p* = 0.001), and at final follow-up were 23.08 ± 3.12 vs. 28.57 ± 3.36 (*p* < 0.05) (Fig. 3, Table 2).

### Radiological outcomes

Radiological outcomes ware comparable and showed no significant difference in preoperative radiological parameters between the two groups. The ROM and the intervertebral disc height of TTL group and Rigid group are shown in Table 3.

**Table 3:** Radiological outcomes

Groups	Preoperative	3 months after operation	1 year after operation	Final follow-up	*P* value
ROM of surgical segment (°)					
TTL (*n* = 24)	8.62 ± 0.75	2.26 ± 0.48	2.83 ± 0.47	3.61 ± 0.65	0.000
Rigid (*n* = 21)	8.49 ± 0.64	1.84 ± 0.33	1.65 ± 0.33	1.44 ± 0.29	0.000
*P* value	0.656	0.002	0.000	0.000	
ROM of upper adjacent segment (°)					
TTL (*n* = 24)	6.78 ± 1.26	7.25 ± 1.14	7.64 ± 1.02	8.51 ± 1.07	0.000
Rigid (*n* = 21)	7.07 ± 1.15	7.69 ± 0.97	8.25 ± 0.85	9.32 ± 0.86	0.000
*P* value	0.546	0.279	0.062	0.012	
DH of surgical segment (mm)					
TTL (*n* = 48)	8.26 ± 1.15	8.26 ± 1.14	8.16 ± 1.18	7.93 ± 1.28	0.175
Rigid (*n* = 42)	8.41 ± 1.10	8.48 ± 1.05	8.03 ± 0.98	7.47 ± 0.91	0.000
*P* value	0.457	0.359	0.472	0.068	
DH of upper adjacent segment (mm)					
TTL (*n* = 24)	10.72 ± 0.93	10.69 ± 0.93	10.16 ± 0.95	9.45 ± 0.90	0.000
Rigid (*n* = 21)	10.87 ± 0.75	10.84 ± 0.79	10.15 ± 0.77	8.94 ± 0.74	0.000
*P* value	0.545	0.569	0.900	0.062	
LL (°)					
TTL (*n* = 24)	27.25 ± 4.41	36.25 ± 3.96	37.25 ± 3.45	38.04 ± 3.74	0.000
Rigid (*n* = 21)	25.43 ± 3.50	36.43 ± 3.97	37.04 ± 4.34	37.57 ± 4.04	0.000
*P* value	0.135	0.873	0.909	0.715	
Modified Pfrrmann grading of surgical segment					
TTL (*n* = 48)	5.67 ± 1.08	N	N	6.00 ± 1.71	0.205
Rigid (*n* = 42)	5.45 ± 0.59	N	N	6.36 ± 0.79	0.000
*P* value	0.212			0.076	
Modified Pfrrmann grading of upper adjacent segment					
TTL (*n* = 24)	3.62 ± 0.49	N	N	4.54 ± 0.51	0.000
Rigid (*n* = 21)	3.71 ± 0.46	N	N	4.62 ± 0.50	0.000
*P* value	0.531			0.604	

*ROM* Range of motion, *DH*, Disc height, *LL* L1–S1 lumbar lordosis angle

There was no significant difference in the ROM and intervertebral disc height of the surgical segment and the upper adjacent segment between TTL group and Rigid group before surgery (*p* = 0.656, *p* = 0.546). At the final follow-up, the ROM of the surgical segment decreased significantly in both groups (*p* < 0.05). There was significant difference in the ROM of the surgical segment between TTL group and Rigid group at postoperative 3-month and1-year and at final follow-up (*p* < 0.05), and that of the upper adjacent segment between TTL group and Rigid group at final follow-up (*p* = 0.012). Additionally, at the postoperative 3-month and 1-year follow-ups, there was no discernible difference in the ROM of the upper adjacent segment between the TTL group and the Rigid group (*p* = 0.279, *p* = 0.062).

At the final follow-up, there was no significant difference in the intervertebral disc height of the surgical segment and upper adjacent segment between the TTL and Rigid groups (*p* = 0.068, *p* = 0.062). The Rigid group's intervertebral disc height of the surgical segment showed significant differences between pre-operation and final follow-up (*p* < 0.05). However, there were no significant differences in the intervertebral disc height of the surgical segment in the TTL group between pre-operation and final follow-up (*p* = 0.175).

The mean LL of TTL group was 27.25° ± 4.41°and that of Rigid group was 25.43° ± 3.50° before surgery (*p* = 0.135). The mean LL of TTL group were 36.25° ± 3.96°, 37.25° ± 3.45° and 38.04° ± 3.74° at postoperative 3-month and1-year and at final follow-up. And the mean LL of Rigid group were 36.43° ± 3.97°, 37.04° ± 4.34° and 37.57° ± 4.04° at postoperative 3-month and1-year and at final follow-up. At each postoperative follow-up time point, there were no statistically significant differences in the mean LL between the TTL group and the Rigid group (*p* = 0.873, *p* = 0.909, *p* = 0.715). There were significant differences of mean LL of each group between pre- and postoperative follow-up. All the patients could maintain their LL at 4-year follow-up.

The modified Pfirrmann grading changes in the surgical segment and the upper adjacent segment in TTL group and Rigid group are shown in Table 3. There was no significant difference between the two groups in the preoperative modified Pfirrmann grading of the surgical segment and the upper adjacent segment (*p* = 0.212, *p* = 0.531). And there was also no significant difference between the two groups in the final follow-up modified Pfirrmann grading of the surgical segment and the upper adjacent segment (*p* = 0.076, *p* = 0.604). The modified Pfirrmann grading changes in the surgical segment of TTL group, there was no significant difference between pre-operation and final follow-up (*p* = 0.205). However, the modified Pfirrmann grading changes in the surgical segment of Rigid group**,** there was significant difference between pre-operation and final follow-up (*p* < 0.05), the modified Pfirrmann grading changes in the upper adjacent segment of both groups, there was also significant difference between pre-operation and final follow-up (*p* < 0.05).

The comparison of dynamic and fusion segments in the Hybrid TTL group are shown in Table 4. Before surgery, there was no significant difference in the ROM of the dynamic and fusion segments (*p* = 0.674), but at the final follow-up, the ROM of both segments was lower than preoperatively (*p* < 0.05). And at the final follow-up, the DH and the modified Pfrrmann grading of the fusion segment was lower than preoperatively (*p* = 0.018, *p* = 0.021).

**Table 4:** Radiological outcomes of TTL hybrid group

Segment (*n* = 24)	Preoperative	3 months after operation	1 year after operation	Final follow-up	*P* value
ROM (°)					
dynamic	5.93 ± 1.19	1.31 ± 0.28	2.05 ± 0.28	2.40 ± 0.43	0.000
fusion	6.01 ± 1.46	0.93 ± 0.21	0.81 ± 0.27	0.60 ± 0.44	0.000
*P* value	0.674	0.000	0.000	0.000	
DH (mm)					
dynamic	9.17 ± 0.69	9.18 ± 0.67	9.12 ± 0.65	9.00 ± 0.64	0.404
fusion	7.36 ± 0.71	7.34 ± 0.69	7.19 ± 0.71	6.85 ± 0.71	0.018
*P* value	0.000	0.000	0.000	0.000	
Modified Pfrrmann grading					
dynamic	4.96 ± 0.86	N	N	5.12 ± 0.54	0.424
fusion	6.37 ± 0.77	N	N	6.88 ± 0.68	0.021
*P* value	0.000			0.000	

*ROM* Range of motion, *DH* Disc height

According to the UCLA classification, the incidence of ASD was 4.2% in the TTL group and 23.8% in the Rigid group, and the incidence of ASD was lower in the TTL group than in the Rigid group (*P* < 0.05, Table 5).

**Table 5:** UCLA system evaluation of intervertebral space of adjacent segment (*n* = 45)

Segment	TTL (*n* = 24)	Rigid (*n* = 21)
L2/3	1(4.2%)	2(9.5%)
L4/5	0 (0%)	3(14.3%)

*P* values are based on the χ2 test. *P* < 0.05 indicates a statistically significant difference

## Discussion

Dynamic stabilization of the lumbar spine is designed to effectively maintain the stability of the spine while preserving a certain degree of physiological mobility in the surgical segment. Furthermore, the dynamic stabilization system has the advantage of limiting the abnormal activity of the surgical segment of the lumbar spine and reducing the excessive mechanical load on the posterior column structure of the lumbar spine while maintaining a certain degree of mobility of the surgical segment, as well as avoiding to some extent the abnormal distribution of stress in the adjacent segments of the disc, thereby effectively reducing the incumbency [11, 12]. The surgical segment load transfer center of the Isobar TTL system is close to the anterior middle column of the spine, similar to the physiological state, and the TTL system is subjected to less compressive stress than conventional rigid fixation devices while still allowing the disc of the operated segment to be subjected to a certain stress load [13]. Some studies have found that when the spinal unit's instability is reduced to a certain extent, this compressive stress load can lead to spontaneous repair of the intervertebral disc [14–16].

In our study, the VASback and VASleg of two groups improved significantly at the final follow-up. And the ODI was also improved significantly in both groups at the final follow-up, which could be attributed to both groups effectively improving the lumbar lordosis angle and restoring a certain lumbar physiological curvature. However, at the last follow-up, the TTL group had lower ODI scores than the Rigid group (26.50 ± 5.48 vs. 30.19 ± 4.64, *p* = 0.02), with the differences primarily in four areas that were strongly associated with improved quality of life: lifting, social life, dressing, and walking. Thus, the results of the TTL group were similar to those of the Rigid group, and in some respects, the clinical symptoms of patients better than those of the Rigid group. Furthermore, the TTL group required less operation time and intraoperative blood loss than the Rigid group (Table 1), which was related to the fact that dynamic fixation of the segment in the TTL group did not require bone grafting and that the device placement process was less disruptive to soft and bone tissues compared to the Rigid group, which is also consistent with the results of our previous meta-analysis [17].

The primary goal of dynamic fixation system research is to preserve segmental mobility, which has a significant impact on ASD prevention. In this study, the mobility of the surgical segment was significantly lower in the TTL group than in the Rigid group at the final follow-up (3.61° ± 0.65° vs. 1.44° ± 0.29°). It is worth noting that as follow-up time increased, so did the segmental mobility of both the fusion and dynamic segments in the TTL group. We suspected that this was due to the postoperative instruction in low back muscle exercise and the adaptive changes in the TTL dynamic device. Given the ODI and segmental mobility differences between the two groups, we speculate that the higher segmental mobility in the TTL group compared to the Rigid group might be one of the reasons why the ODI score was considerably lower in the TTL group at the final follow-up.

**Fig. 5:**
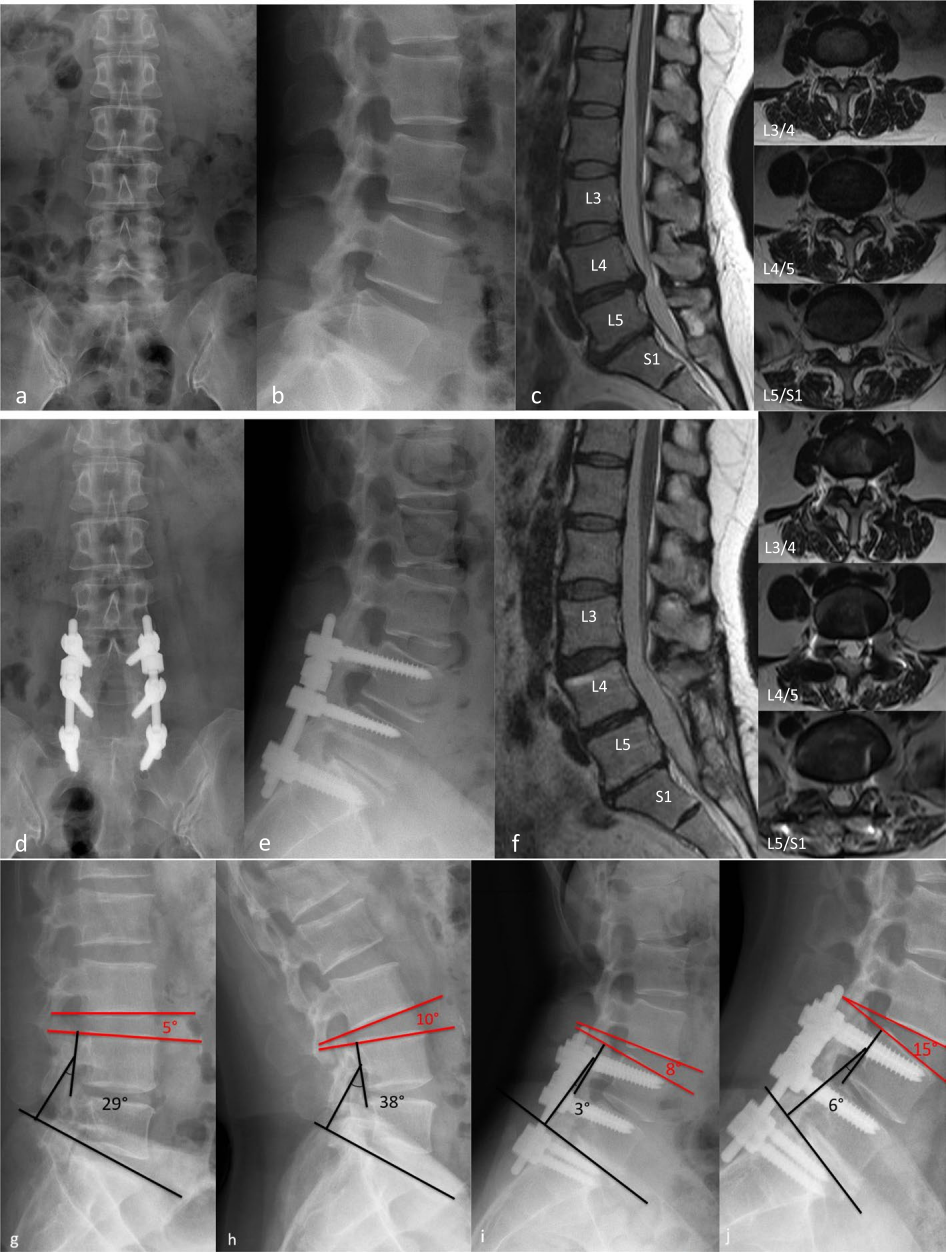
A 59-year-old male patient underwent Isobar TTL hybrid fixation for ASD prevention(L4/S1). **a–c** Preoperative radiological imaging confirmed L4/5 spinal stenosis and disc herniation, and L5/S1 with narrowing of the intervertebral space, calcification of the ligamentum flavum, and spinal stenosis. **d–f** L4/5 for mildly degeneration with dynamic fixation, Since the L5/S1 segment degeneration is more severe than that of the L4/5 segment, posterolateral fusion is used. Radiological imaging 27 months after surgery indicated that satisfactory decompression was achieved. **g-h** Preoperative radiological imaging showed that ROM of upper adjacent segment and operative segment is 5° and 7° respectively. **i-j** Final follow-up radiological imaging showed that ROM of upper adjacent segment and operative segment is 9° and 3° respectively

Another main goal of the dynamic stabilization system in the treatment of mild and moderate lumbar degenerative disease is to reduce compensatory mobility in the adjacent phases in order to reduce the incidence of ASD. The mechanical feasibility has been demonstrated in finite element studies [18, 19], but there is still much debate about whether it can reduce the incidence of ASD in clinical practice, as well as its medium- and long-term clinical efficacy [20, 21]. In our study, the mobility of the upper adjacent segment increased significantly in both groups compared to the pre-operation at the final follow-up, but the compensatory mobility of the upper adjacent segment was significantly lower in the TTL group than in the Rigid group, indicating that the TTL system was more effective than rigid fixation in reducing compensatory mobility of the upper adjacent segment (Figs. 4 and 5).

Disc height is an important indicator of disc degeneration, some studies have shown that the disc height increases after dynamic fixation, but gradually decreases from long-term follow-up [22]. In our study, there was no significant difference in the DH of the surgical segment 3 months after surgery in either group as compared to pre-operation. Despite a decreasing tendency in both groups as follow-up time increased, the difference in DH of the TTL group at the last follow-up compared to preoperative was not statistically significant (Table 4). The modified Pfrrmann grading of the surgical segment increased at the final follow-up in both groups. The difference at the final follow-up compared to the preoperative period, however, was not statistically significant in the TTL group, but it was in the Rigid group. In the TTL group, we also found no significant difference in dynamic segmental DH at the final follow-up compared to preoperative, while fusion segments were significantly lower than preoperative. And the modified Pfrrmann grading of the dynamic segments were not statistically significant at the last follow-up compared to preoperative, while fusion segments were statistically significant. These suggest that TTL has a preventive effect on the degeneration of surgical segmental discs. We concluded that this could be because the TTL system's dynamic segmentation allows the disc to still be subjected to a certain stress load, and this compliance prevents the stress masking effect in rigid fixation, which is important for disc self-recovery. Furthermore, in our experience, when the annulus fibrosus is not fractured, surgery without removing the disc can be performed, and adequate neurological decompression can be obtained by removing the laminae, facets, and ligamentum flavum, which may also be one of the reasons for the lower degree of disc degeneration in dynamic segments compared to fusion segments.

For the upper adjacent segment, DH was significantly lower in both groups at the final follow-up compared to the pre-operation, but there was no significant difference between groups, which was consistent with the findings of Yu et al. [23] and Fei et al. [24]. However, this study also discovered a tendency for DH to decrease over time in both groups, and group differences may emerge gradually with longer follow-up time. Furthermore, UCL grading revealed that one upper adjacent segment (4.2%) in the TTL group and five upper adjacent segments (23.8%) in the Rigid group regressed, indicating that the incidence of ASD was higher in the Rigid group than in the TTL group, implying that the Isobar TTL dynamic system slowed the occurrence of ASD to some extent. However, the modified Pfrrmann grading of the upper adjacent segment was significantly higher at the final follow-up, indicating disc degeneration in both groups. As a result, it remains to be seen whether the TTL system for the Hybrid procedure can reduce the incidence of ASD by preserving surgical segment mobility and reducing pressure on the intervertebral joint and compensatory activity of the adjacent segment [25].

Although previous research has shown that repetitive internal stresses can cause screw loosening in dynamic fixation versus rigid fixation [26], no screw loosening cases were found in our study. This could be because the Isobar TTL system's surgical segment load transfer center is close to the anterior and middle columns of the spine, putting less compressive stress on the nails and rods than other dynamic fixation devices. Furthermore, we believe that this is closely related to the precise placement of the nail and the preservation of the synovial joint during decompression to maintain as much spine stability as possible.

The current study demonstrated the two-level Isobar TTL hybrid system's safety and effectiveness. Though the surgical levels showed image evidence of disc rehydration, the prevention effect of ASD remained unknown. More research is needed to confirm the impact on ASD.

## Conclusion

We attempted to apply their many years of experience in dynamic lumbar fixation to a two-level lumbar hybrid procedure, and no obvious signs of lumbar instability were seen on postoperative follow-up radiographs. Overall clinical efficacy was comparable to titanium rod fusion surgery during the same period, and intervertebral disc protection was more advantageous than titanium rod fusion surgery, providing an alternative treatment option for mild and moderate lumbar degenerative diseases. Additional research with large sample sizes and multiple centers is required to determine the impact on adjacent segments.

## Data Availability

The datasets used and/or analyzed during the current study are available from the corresponding author on reasonable request.
